# High-Sensitivity 2D MoS_2_/1D MWCNT Hybrid Dimensional Heterostructure Photodetector

**DOI:** 10.3390/s23063104

**Published:** 2023-03-14

**Authors:** Nanxin Fu, Jiazhen Zhang, Yuan He, Xuyang Lv, Shuguang Guo, Xingjun Wang, Bin Zhao, Gang Chen, Lin Wang

**Affiliations:** 1 School of Materials and Chemistry, the University of Shanghai for Science and Technology, Shanghai 200093, China; 2 State Key Laboratory of Infrared Physics, Shanghai Institute of Technical Physics, Chinese Academy of Sciences, Shanghai 200083, China

**Keywords:** oriented multi-walled carbon nanotubes, molybdenum disulfide, mixed dimensions, heterostructures, photodetectors

## Abstract

A photodetector based on a hybrid dimensional heterostructure of laterally aligned multiwall carbon nanotubes (MWCNTs) and multilayered MoS_2_ was prepared using the micro-nano fixed-point transfer technique. Thanks to the high mobility of carbon nanotubes and the efficient interband absorption of MoS_2_, broadband detection from visible to near-infrared (520–1060 nm) was achieved. The test results demonstrate that the MWCNT-MoS2 heterostructure-based photodetector device exhibits an exceptional responsivity, detectivity, and external quantum efficiency. Specifically, the device demonstrated a responsivity of 3.67 × 10^3^ A/W (λ = 520 nm, V_ds_ = 1 V) and 718 A/W (λ = 1060 nm, V_ds_ = 1 V). Moreover, the detectivity (D*) of the device was found to be 1.2 × 10^10^ Jones (λ = 520 nm) and 1.5 × 10^9^ Jones (λ = 1060 nm), respectively. The device also demonstrated external quantum efficiency (EQE) values of approximately 8.77 × 10^5^% (λ = 520 nm) and 8.41 × 10^4^% (λ = 1060 nm). This work achieves visible and infrared detection based on mixed-dimensional heterostructures and provides a new option for optoelectronic devices based on low-dimensional materials.

## 1. Introduction

Low-dimensional semiconductor materials are a class of materials that emerged rapidly in the 20th century with the development of nanoscience and technology [1]. Typical low-dimensional semiconductor materials can be classified by dimensionality into two-dimensional superlattices and quantum wells, one-dimensional nanowire materials, and zero-dimensional quantum dots. In addition, various 1D nanostructures, such as semiconductor nanowires, nanoribbons, nanorods, and nanotubes, have been successfully manufactured. As a result, there has been a global surge in research activity focused on exploring the unique properties and potential applications of these materials [2,3,4,5,6,7]. Numerous studies have demonstrated the potential of these one-dimensional (1D) nanomaterials and structures for applications in nano-optoelectronics and electronic devices [8,9,10,11,12,13,14,15,16]. In recent years, carbon nanotubes (CNTs) have garnered considerable attention from the scientific community, owing to their remarkable intrinsic properties. With their ultrathin mass (1–3 nm), CNTs are seen as an ideal material for use as a channel or active material in nanoelectronics and optical electronics. The material’s ballistic transport, high stability, and mobility further contribute to its suitability for these applications. These unique properties of CNTs have made them a material of great interest for scientists and engineers, who are constantly exploring their potential applications in various fields [17,18,19,20]. Furthermore, how CNTs are convoluted by graphene sheets strongly determines their electronic properties [21]. Carbon nanotubes (CNTs) exhibit unique electrical behavior that can be either metallic or semiconducting depending on their chirality angle or helicity index [22]. Multi-walled carbon nanotubes (MWCNTs) are often characterized by a lack of regularity in terms of tube shape, as well as an unequal distribution of layers and disorderly arrangement. In the realm of carbon-nanotube-based photodetectors, thin films have been the material of choice. However, it is important to note that the orientation, uniformity, and surface defects of these films have a direct impact on the performance of the resultant devices. Therefore, achieving a high degree of control over the morphology and structure of MWCNTs and carbon nanotube thin films is of critical importance to the development of high-performance photodetectors. Two-dimensional (2D) materials offer distinct advantages due to the absence of surface hanging bonds. As a result, the complexification of surface structures can be effectively suppressed, leading to an enhanced stability and reproducibility of their physical and chemical properties. This makes 2D materials highly attractive for a broad range of applications, including electronics, photonics, energy storage, and catalysis. Furthermore, the unique mechanical, optical, and electronic properties of 2D materials also make them highly promising candidates for use in novel nanoscale devices and systems [23,24,25,26]. MoS_2_ is one of the most widely studied materials in 2D layer TMDs, and exhibits unique electronic, optical, and mechanical properties due to its atomically thin structure. However, the characteristics of MoS_2_ limit visible light absorption, and the limitation of the separation of photogenerated carriers by the significant exciton effect of two-dimensional materials makes parameters such as the optical responsivity of MoS_2_ photodetectors more limited, which hinders its development in the direction of high-performance photodetectors. Fortunately, although light absorption is an inherent property of the material, the construction of heterojunctions can effectively complement the light absorption of a single MoS_2_ material and enhance the overall light absorption. In addition, the carrier mobility can be effectively improved by constructing heterojunctions to enhance the optical response rate. Thus, it is a promising material for forming heterostructures with CNTs. In recent years, numerous studies have been aimed at enhancing the efficiency of MoS_2_ photodetectors by means of a systematic design of van der Waals heterostructures [27,28,29,30].

Through van der Waals force binding, a one-dimensional (1D) and a two-dimensional (2D) material can form unique mixed-dimensional heterostructures that inherit the unique physical properties of van der Waals 2D/2D heterostructures, but also have rich stack configurations, providing new manipulable freedom to adjust the structure and properties of heterostructures [31,32,33,34,35,36]. The versatility of heterostructure fabrication provides researchers with a new level of control over the resulting structure and properties. Such heterostructures have the potential to enable the creation of new and unique electronic and optoelectronic devices. Considering the previous reports of CNT/MoS_2_ heterostructures for photodetectors [37,38,39,40], most of them are based on randomly aligned carbon nanotube networks. However, few studies have used high-quality carbon nanotubes with highly directional alignment, most of the detection range is limited to visible light, and no broad spectrum detection from visible to near infrared has been achieved. The implementation of highly aligned carbon nanotubes in heterostructure fabrication could provide significant advantages, such as an improved structural stability and increased device performance. The ability to tailor the structure and properties of these heterostructures could lead to a further optimization of their detection capabilities for specific applications. Furthermore, achieving broad-spectrum detection from visible to near-infrared wavelengths would be highly desirable for many applications, including imaging and sensing technologies. Thus, there is a significant opportunity for further research in the development and optimization of CNT/MoS_2_ heterostructures for photodetectors.

This paper reports a photodetector device based on a lateral hybrid vdW heterostructure composed of directionally aligned multi-walled CNT bundles and MoS_2_. The introduction of CNTs in the vdW heterostructure reduces the contact area to the nanometer scale. The MWCNT-MoS_2_ planar heterostructures were characterized by Raman spectroscopy, Raman mapping, and AFM. In addition, the MWCNT-MoS_2_ back-gate FETs have rectification characteristics. The increased light absorption by the heterostructure and the integrated electrical field contribute to improving the photoresponse and detection rate of the photodetector. The device exhibits a distinct photoresponse, and the photocurrent magnitude grows with power density under laser irradiation at 520 nm and 1060 nm, respectively. This work provides the basis for the study of the electrical and optoelectronic properties of hybrid vdW heterostructure devices, which demonstrate the possibility of manufacturing 1D–2D hybrid structures for future nanoelectronics and nanophotonics.

## 2. Materials and Methods

### 2.1. Preparation of 1D CNT Bundles

**Figure 1:**
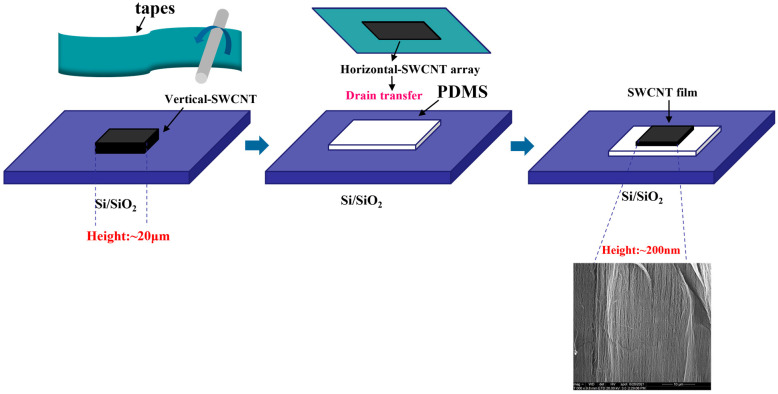
Schematic depicting the fabrication process for CNT bundles in a laterally aligned formation; the inset shows the SEM image of the processed CNT bundles.

The height of the CVD-grown CNT arrays was approximately 20 μm. The as-obtained material was prepared into a thin film structure by rolling the sample with a smooth roller under certain pressure conditions and then transferring it onto a tape. In this way, it can be transformed into a horizontal alignment from vertical alignment, transferred onto a substrate (Si substrate with 285 nm SiO_2_ layer) using a dry method, and then transferred onto a slide affixed with PDMS using a dry method [41,42]. The fabrication process for CNT bundles in a laterally aligned formation is shown in Figure 1.

### 2.2. Preparation of Thin Layers of Molybdenum Disulfide

First, a block of molybdenum disulfide material with good monocrystalline was pre-pared, a flatter, smoother, glossy piece of material was glued from the block with the help of blue tape, and a thin layer of molybdenum disulfide was obtained by mechanical peeling method, which was then transferred to a soft polydimethylsiloxane (PDMS) substrate on a transparent slide to find the right thickness of material under the microscope.

### 2.3. Materials Characterization

The SEM images were obtained by ZEISS Sigma 300 Field Emission Scanning Electron Microscope, Raman imaging, and Raman spectra were achieved by LabRAM ODYSSEY using a 532 nm laser as an excitation source. Polarized Raman spectra were obtained at the 514.5 nm laser equipped with an objective of 100× as the excitation source. Material thickness and surface roughness were obtained by AFM. The phase of the MWCNT and MoS_2_ materials was determined through ultra-high resolution X-ray diffraction (XRD) measurements using a Bruker-AXS instrument.

### 2.4. Devices Fabrication and Measurement

**Figure 2:**
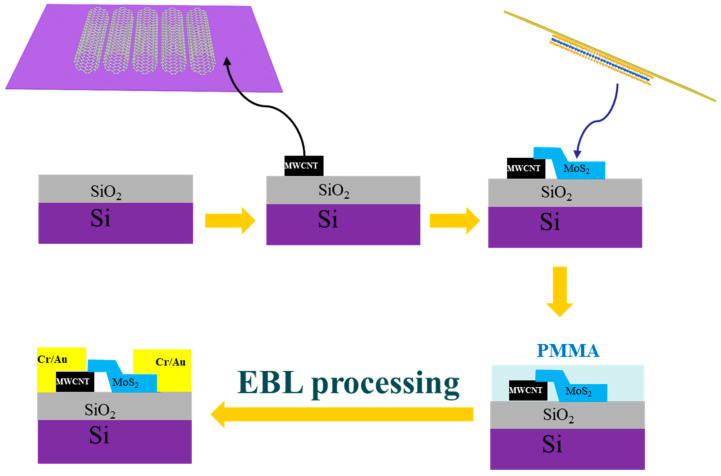
Schematic diagrams depicting the fabrication procedure for lateral vdW heterostructure.

The fabrication procedure for lateral vdW heterostructure is shown in Figure 2. A suitable thickness of MoS_2_ was transferred to MWCNTs using a micro-nano spot transfer technique [43], and then a standard lithography technique was used to etch a painted electrode pattern onto the target wafer by electron beam exposure (EBL), followed by electron beam deposition of Cr/Au (thickness: 20 nm/80 nm) and a subsequent peeling process to prepare gate FETs based on MoS_2_ and MWCNT-MoS_2_ heterostructures. The electrical and optoelectronic tests were performed using Lake Shore TTPX probe station and Keithley 4200 semiconductor device parameter analyzer. The incident light had wavelengths of 520 nm and 1060 nm and the spot size was approximately 1 mm^2^. All optoelectronic tests were measured at room temperature in the air.

## 3. Results and Discussion

**Figure 3:**
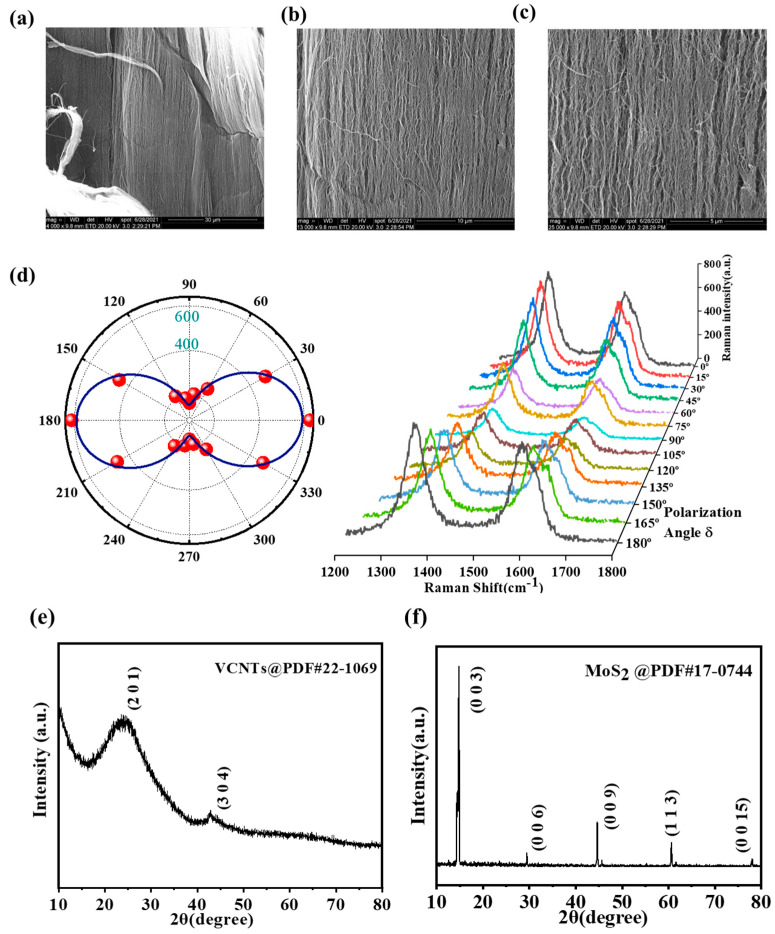

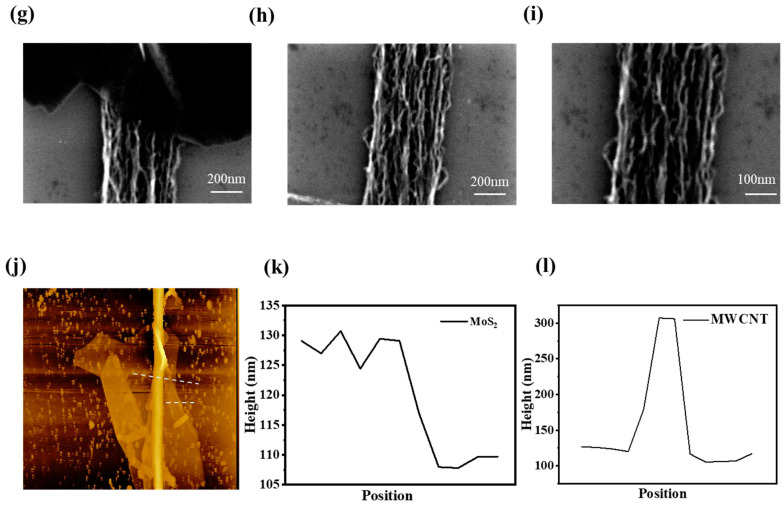
(**a**–**c**) The SEM images of the obtained MWCNTs with different magnifications; (**d**) the polar plot of the intensity of Raman G-peaks of MWCNTs vs. φ; (**e**,**f**) XRD pattern of VCNTs and MoS_2_ bulk material; (**g**) the SEM image of MWCNT-MoS_2_ heterostructure, (**h**,**i**) the SEM images of CNT at the heterostructure junction with different magnifications; (**j**) AFM image of the heterostructure; (**k**,**l**) thickness of the edge MoS_2_ region and the planar stacked MWCNT, respectively.

The obtained MWCNTS were first characterized by SEM—the SEM images in Figure 3a–c show a highly ordered arrangement of CNTs—and then we tested them with Raman polarization spectroscopy. The polar diagram of the Raman G-peak maximum intensity of the CNT bundles given in Figure 3d similarly confirms the orientation of the CNT that we obtained [44]. To confirm the phases of the vertically grown multi-walled carbon nanotube (MWCNT) arrays and MoS_2_ material, X-ray diffraction (XRD) tests were carried out, and the results are shown in Figure 3e,f. The obtained XRD pattern of the CNT conforms to the standard card PDF #22-1069, where the main peaks of the bare CNT appear at 2θ = 23.9° and 43.0°, corresponding to its (201) and (304) planes, respectively. As for the MoS_2_ bulk material, the XRD pattern exhibits peaks at 14.5°, 29.3°, 44.5°, 60.5°, and 77.9°, which can be ascribed to the (003), (006), (009), (113), and (0015) planes, respectively, when compared to the standard card PDF #17-0744. Figure 3g shows the image of the obtained heterogeneous structure under scanning electron microscopy (SEM) at different magnifications. Figure 3j shows the AFM characterization of this sample, and the results show that the surface of the sample is relatively homogeneous, where the thickness of MoS_2_ is approximately 20 nm for multilayer MoS_2_, whereas the thickness of MWCNT is approximately 200 nm.

**Figure 4:**
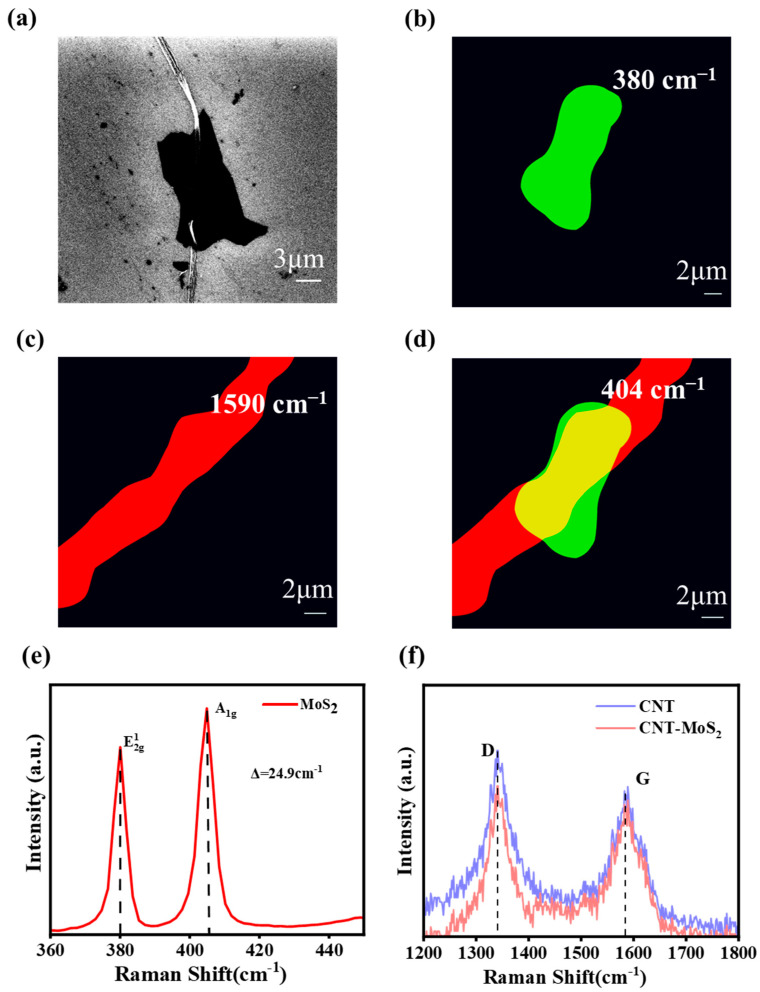
(**a**) Scanning electron microscopy images of selected individual MWCNT-MoS_2_ heterostructure; (**b**–**d**) Raman mapping acquired from the MoS_2_ region and the planar stacked MWCNT-MoS_2_ region, respectively; (**e**) Raman spectra of MoS_2_; (**f**) Raman spectra of CNT-MoS_2_ and bare CNT.

To demonstrate the construction of the MWCNT-MoS_2_ heterostructure, a series of characterizations of the obtained samples were carried out. First, the Raman mapping of the samples is given in Figure 4, where Figure 4a shows the SEM photographs of the test samples and Figure 4b–d show the Raman peak intensity mapping of the samples at 380 cm^−1^, 404 cm^−1^, and 1590 cm^−1^ wave numbers, respectively. The peak intensity of the sample is at the 404 cm^−1^ wave number (representing the MoS_2_ characteristic peak) mapping signal, and the peak of MoS_2_ appears in the region and MWCNT-MoS_2_ region. There are two peaks located at 380 cm^−1^ and 404.9 cm^−1^ (Figure 4e), corresponding to the in-plane E^1^_2g_ and A_1g_ vibration of MoS_2_, respectively, indicating that the MoS_2_ is multilayer [45]. In addition, the intensity ratio of the MWCNT Raman G-peak to the D-peak is slightly increased, which is attributed to the formation of the heterostructure. Combined with the SEM image results, the MWCNT-MoS_2_ van der Waals heterostructure was successfully constructed in the central region of the sample. In addition, the Raman mapping of the sample has a more uniform color lining, which indicates that the sample has good homogeneity in the two-dimensional plane.

**Figure 5:**
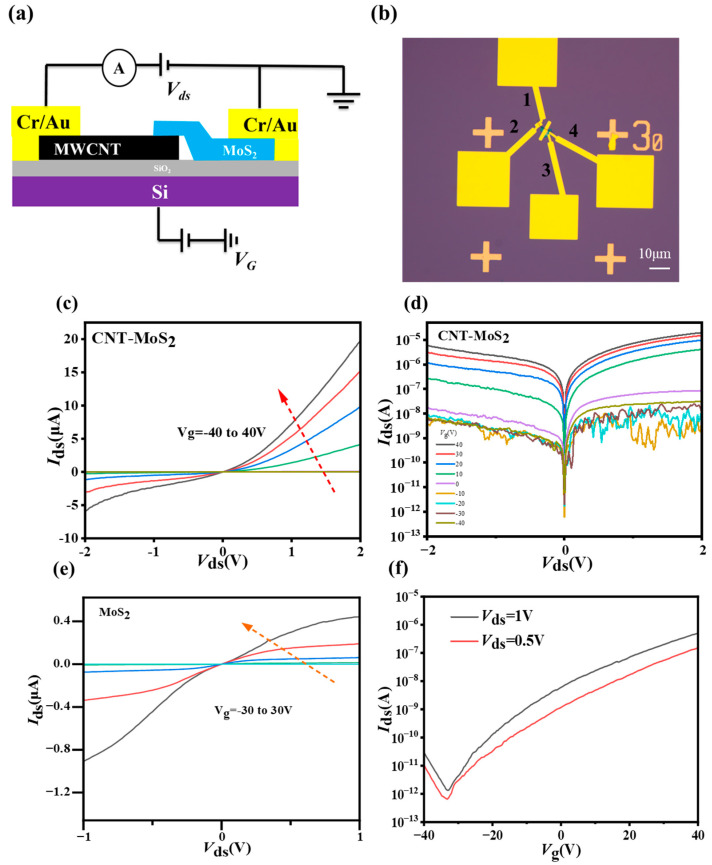
(**a**) Schematic diagram of a representative device; (**b**) optical image of the device with electrodes 1 and 2 in contact with CNT and electrodes 3 and 4 in contact with MoS_2_; (**c**) transfer characteristics of the heterostructure devices at different gate voltages; (**d**) corresponding logarithmic plots of the transfer curves; (**e**) transfer characteristics of MoS_2_ device at different gate voltages; (**f**) transfer curves of MoS_2_ at 0.5 V and 1 V bias voltage, respectively. Notes: ((**c**,**e**) the missing curves overlap at the 0 axis).

In order to investigate the effect of heterogeneous interfaces on charge transport behavior, gate FETs based on a bare MoS_2_ and MWCNT-MoS_2_ heterostructure were constructed using standard photolithography techniques. The optical photograph of the device is shown in Figure 5b, where the MWCNT-MoS_2_ heterostructure FET is constituted between electrodes 1 and 3 or 2 and 4, and the bare MoS_2_ FET is constituted between electrodes 3 and 4.

Figure 5c gives the transfer characteristic curves of the device and Figure 5d shows the corresponding logarithmic curve of its transfer curve. The transfer curves of the device show that, when the source-drain voltage V_ds_ of the device is negative, the I_ds_ of the device change very little with an increasing voltage, whereas, when the V_ds_ of the device is positive, the value of the I_ds_ of the device increases rapidly with an increasing voltage, which shows that the current rectification characteristic is similar to that of a diode.

To further demonstrate that the rectification characteristics are caused by the p-n heterojunction, the electrical properties of MoS_2_ devices were tested. Figure 5e gives the transfer characteristic curves of MoS_2_ devices at different gate voltages in the source/drain bias voltage range −1~1 V. The current increases with an increasing V_g_ and the curves exhibit a typical n-type behavior. In contrast, the transfer curves of heterojunction FETs exhibit typical p-n junction conductivity behavior. Therefore, the rectification characteristics of the CNT-MoS_2_ heterojunction FET are due to the p-n heterojunction, since the device forms a p-n heterojunction that leads to forward conductivity and good correction characteristics.

**Figure 6:**
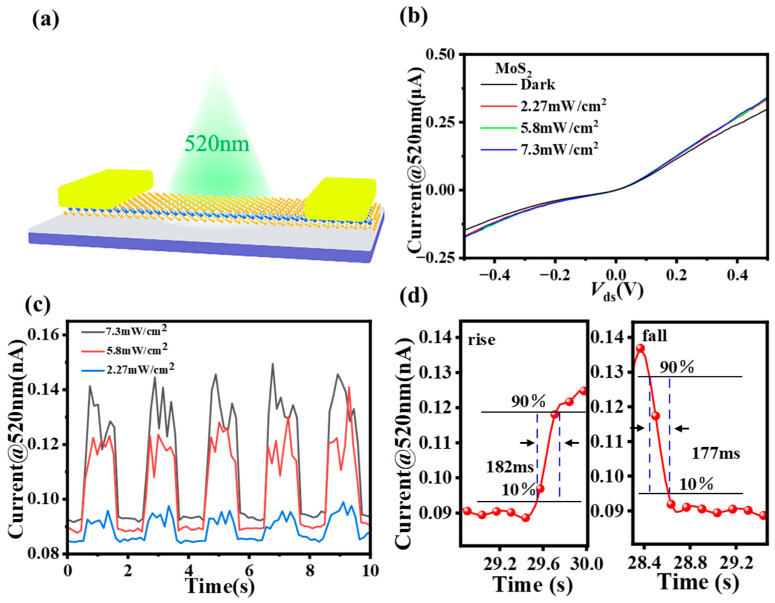
(**a**) Schematic illustration of the bare MoS_2_ photodetector; (**b**) I_ds_-V_ds_ curves of the bare MoS_2_ photodetector at V_g_ = 0 V with different laser powers at V_ds_ = 0 V; (**c**) I_ds_-V_ds_ curves of the bare MoS_2_ photodetector at V_g_ = 0 V with different laser powers at V_ds_ = 0.01 V; (**d**) variation in the device response time at V_ds_ = 0.01 V with an optical power of 5.8 mW/cm^2^ illumination.

To further determine the optical response of the device, a broad-beam laser with an adjustable power density was used as the light source to measure the current-time (I-T) characteristics under different power density irradiation. We compared the photoresponse variations in the bare MoS_2_ photodetector and heterostructure devices and all experiments were performed in a room-temperature environment. The response time (defined as the increase in photocurrent from 10% to 90% of the peak current) is shown in Figure 6d for the bare MoS_2_ photodetector, from which, it can be seen that the photoresponse of the bare MoS_2_ photodetector is not very significant and the response time is slow, with a rise time of 182 ms and a fall time of 177 ms. *R_I_* is the most commonly used parameter for characterizing the sensitivity of a photodetector, defined as the photocurrent induced per unit power irradiated on the photosensitive surface of the photodetector, and its equation can be expressed as:

*R_I_* = *I_ph_/P_in_* ∗ *S* (1)

where *P_in_* is the optical power density, *S* is the effective area of the device under illumination, and *I_ph_* is the corresponding photocurrent. The effective light area of the MWCNT-MoS_2_ photodetector and the bare MoS_2_ photodetector are approximately 0.6 μm^2^ and 6 μm^2^, respectively. By calculating, the photocurrent responsivity of the MWCNT-MoS_2_ and bare MoS_2_ photodetector is 183 A/W and 0.11 A/W (P = 2.27 mW/cm^2^, V_ds_ = 0.01 V), respectively. The responsivity value of the MWCNT-MoS_2_ photodetector is much higher than those reported of the photodetectors (Table 1). The data presented in Table 1 clearly illustrate that the choice of the heterojunction structure, material synthesis method, material thickness, and measurement conditions can significantly impact the magnitude of the R. It is worth noting that the present study employs a method that is both straightforward and practical, enabling the achievement of remarkable R-values in MWCNT-MoS_2_ heterostructures.

In our heterostructure device, the maximum photocurrent responsivity is 3.67 × 10^3^ A/W at a wavelength of 520 nm (P = 2.27 mW/cm^2^, V_ds_ = 1 V), and the value is much higher at lower powers. D* indicates the ability to detect weak light signals, and can be expressed as:

(2) $$D*=R\sqrt{S/2qI_{dark}}$$

Based on this equation, D* = 1.2 × 10^10^ Jones is calculated for a heterostructure device at an optical power density of 2.27 mW/cm^2^ at a bias voltage of 1 V. *EQE* is the ratio of the number of photogenerated carriers to the number of incident photons, and can be expressed as:

(3) $$EQE=R_{\lambda }\frac{hc}{e\;\cdot \;\lambda }\times 100\%$$

where *h* is Planck’s constant and *e* and *c* are the fundamental charge and the speed of light, respectively. λ is the wavelength of the incident light. Thus, when *R_I_* = 3.67 × 10^3^ A/W (λ = 520 nm, P = 2.27 mW/cm^2^, V_ds_ = 1 V), the corresponding *EQE* is up to 8.77 × 10^5^%, indicating an excellent performance improvement in our MWCNT-MoS_2_ photodetector.

**Figure 7:**
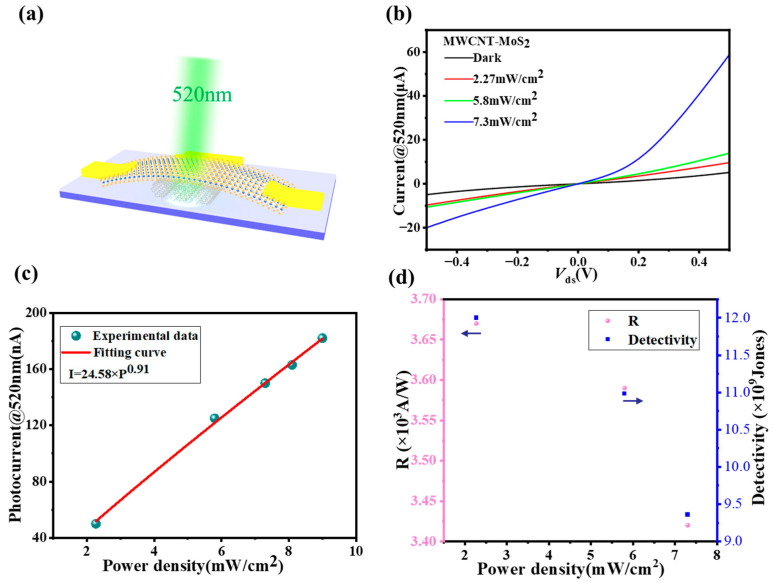

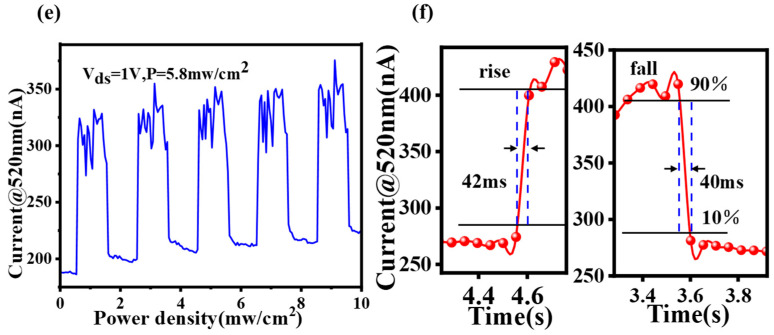
(**a**) Schematic illustration of the heterostructure photodetector; (**b**) I_ds_-V_ds_ curves of the heterostructure photodetector at V_g_ = 0 V and V_ds_ = 0 V with different laser powers; (**c**) photocurrent vs. power density of the MWCNT-MoS_2_ heterostructure photodetector device at V_g_ = 0 V and V_ds_ = 1 V; (**d**) variation in detection rate and responsiveness at different optical power densities; (**e**) variation in device response time at V_ds_ = 1 V with an incident power of 5.8 mW/cm^2^ illumination; (**f**) time-resolved photoresponse, where the rise and fall times of the photocurrent are 42 ms and 40 ms, respectively.

By increasing the power of the incident light at 520 nm, the number of photogenerated carriers in the channel is increased correspondingly, so the magnitude of the photocurrent shows a linear enhancement when increasing the incident light power (Figure 7c), which is consistent with the relationship between *I_ph_* and incident power:

*I_ph_* = *AP^⍺^* (4)

The value of *⍺* obtained by fitting is approximately 0.91, which is very close to the ideal value of 1, indicating that the process is very efficient for electron–hole pair separation and complexation. The optical responsiveness *R_I_* and detection D* of the MWCNT-MoS_2_ heterostructure photodetector with laser power density for V_ds_ = 1 V are given in Figure 7d, and both the optical responsiveness *R_I_* and detection D* decrease with an increasing laser power. By switching the laser periodically, the device can switch between ON and OFF states effectively, and there is no significant current decay, indicating that the MWCNT-MoS_2_ photodetector has good stability. From the device response and reset curves for a single cycle given in Figure 7f, the rising-edge response time τ_rise_ for the detector is 42 ms, and the falling-edge response time τ_fall_ is 40 ms.

**Figure 8:**
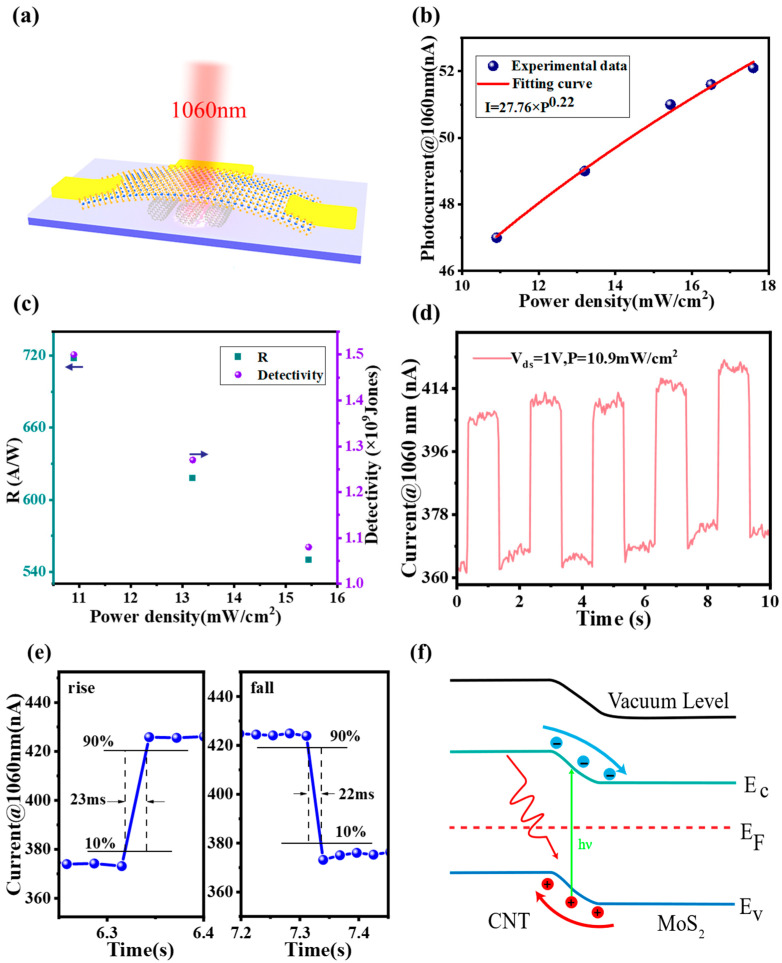
(**a**) Schematic illustration of the device under NIR (1060 nm) laser irradiation; (**b**) photocurrent vs. power density of the MWCNT-MoS_2_ heterostructure photodetector device at V_g_ = 0 V and V_ds_ = 1 V; (**c**) variation in detection rate and responsiveness at different optical power densities; (**d**) variation in device response time at V_ds_ = 1 V with an incident power of 10.9 mW/cm^2^ illumination; (**e**) time-resolved photoresponse, where the rise and fall times of the photocurrent are approximately 23 ms and 22 ms, respectively; (**f**) energy band diagrams of CNT/MoS_2_ vdW heterostructures in the light.

The optoelectronic response of a 1060 nm wavelength laser was also studied, as shown in Figure 8b–e. The maximum photocurrent responsivity is 718 A/W at a wavelength of 1060 nm (P = 10.9 mW/cm^2^, V_ds_ = 1 V), and *EQE* = 8.41 × 10^4^%, D* = 1.5 × 10^9^ Jones. To better understand the heterostructure photodetector response mechanism in the visible–NIR, a schematic diagram of the device’s energy band under light illumination is given in Figure 8f. Under laser irradiation, electron–hole pairs are generated within the MoS_2_ layer. Then, the electrons can be transferred to the surface of MWCNT. Compared with bare MoS_2_ FETs, the heterostructure provides an enhanced photon absorption, and when the laser irradiates the heterostructure, the photogenerated electron–hole pairs are separated at the MWCNT-MoS_2_ heterojunction interface due to the presence of the built-in electric field, generating photocurrents and promoting the capture of photogenerated carriers by the electrodes, speeding up the response so that the device can operate at V_ds_ = 0 V. When a forward bias is applied to the device, the p-n junction is in the open state and the superposition of the internal and external electric fields can separate the photogenerated electron–hole pairs more effectively, enabling the device to obtain a larger photocurrent. However, the device can obtain a higher photocurrent at V_ds_ = 1 V with a concomitant increase in the dark current. Therefore, when a bias voltage of V_ds_ = 1 V is introduced, the detectivity of the device decreases significantly, although the responsiveness of the photodetector can be enhanced. Overall, the performance of the MWCNT-MoS_2_ photodetector is significantly improved compared to the bare MoS_2_ photodetector, and this improved heterostructure system optimizes the performance of the photodetector. Moreover, current instability is observed in both dark and illumination conditions. It is possible that the MWCNT-MoS_2_ heterostructure has an imperfect material contact interface that leads to carrier capture and release, which can result in jitter, such as the capacitive effect [48]. This phenomenon has also been observed in some hybrid dimensional heterostructure photodetectors, as reported in a previous study [49,50]. In the case of the MWCNT-MoS_2_ heterostructure, the carbon nanotube is one-dimensional, and the interface with the two-dimensional material may be less than ideal. However, testing the device in a vacuum environment could significantly reduce the current instability as it would minimize the presence of impurities and other contaminants that may contribute to the carrier capture and release. Nevertheless, further investigation is needed to determine the root cause of the current instability and develop strategies to improve the device’s performance.

## 4. Conclusions

In conclusion, we demonstrated a hybrid dimensional heterostructure based on directionally aligned MWCNTs with MoS_2_ for a high-sensitivity photodetector, which was successfully prepared using a micro-nano fixed-point transfer technique. We found that the heterostructure device can observe stable optical response waveforms in a wide wavelength range from visible (520 nm) to near-infrared (1060 nm), demonstrating a nanomaterial-based heterostructure in which the advantageous properties of the components are combined to substantially improve the device properties. The photodetector provides a clear optical response when irradiated by laser light in the environment, and shows light-sensing capability at various power intensities and wavelengths. The device fabrication method is convenient and easy to control, which makes it a promising candidate for low-cost photodetection.

## Figures and Tables

**Table 1:** Comparison of the responsivity values of MoS_2_ and CNT-MoS_2_ photodetectors.

Device Structure	Measurement Condition	Responsivity	Reference
Multilayer MoS_2_	λ = 532 nm, V_ds_ = 1.2 V, P = 1.69 mW/cm^2^	59 A/W	[46]
s-SWCNT (network)/SL-MoS_2_	λ = 650 nm, V_ds_ = −5 V, V_g_ = −40 V, P = 280 μW	>0.1 A/W	[37]
MWCNT (powder)/MoS_2_ core–shell	λ = 532 nm, V_ds_ = 2 V, P = 1 mW	24 m A/W	[38]
MoS_2_ (fewer-layer)/SWCNTs network	λ = 532 nm, V_ds_ = 3 V, P = 4 μW/cm^2^	8 × 10^3^ A/W	[39]
SWCNT/MoS_2_ (bi- or tri-layer) as-grown heterostructures	λ = 532 nm, V_ds_ = 0.1 V, P = 0.2 × 10^−3^ mW/cm^2^	300 A/W	[40]
SWCNTs/multilayer MoS_2_/ITO	λ = 532 nm, V_ds_ = 1 V, P = 10 nW	2008.3 A/W	[47]
MWCNT (horizontally aligned)/multilayer MoS_2_	λ = 520 nm, V_ds_ = 1 V, P = 2.27 mW/cm^2^	3670 A/W	This work
	λ = 1060 nm, V_ds_ = 1 V, P = 10.9 mW/cm^2^	718 A/W	

## Data Availability

Not applicable.
